# ARF5/MONOPTEROS directly regulates miR390 expression in the *Arabidopsis thaliana* primary root meristem

**DOI:** 10.1002/pld3.116

**Published:** 2019-02-05

**Authors:** Mouli Ghosh Dastidar, Andrea Scarpa, Ira Mägele, Paola Ruiz‐Duarte, Patrick von Born, Lotte Bald, Virginie Jouannet, Alexis Maizel

**Affiliations:** ^1^ Center for Organismal Studies (COS) University of Heidelberg Heidelberg Germany; ^2^ Present address: PsiOxus Therapeutics Abingdon UK; ^3^ Present address: Max Planck Institute for Plant Breeding Research Cologne Germany

**Keywords:** Arabidopsis, auxin, meristem, miRNA, root

## Abstract

The root meristem is organized around a quiescent center (QC) surrounded by stem cells that generate all cell types of the root. In the transit‐amplifying compartment, progeny of stem cells further divides prior to differentiation. Auxin controls the size of this transit‐amplifying compartment via auxin response factors (ARFs) that interact with auxin response elements (AuxREs) in the promoter of their targets. The microRNA miR390 regulates abundance of ARF2, ARF3, and ARF4 by triggering the production of trans‐acting (ta)‐siRNA from the *TAS3* precursor. This miR390/TAS3/ARF regulatory module confers sensitivity and robustness to auxin responses in diverse developmental contexts and organisms. Here, we show that miR390 is expressed in the transit‐amplifying compartment of the root meristem where it modulates response to exogenous auxin. We show that a single AuxRE located in miR390 promoter is necessary for miR390 expression in this compartment and identify that ARF5/MONOPTEROS (MP) binds miR390 promoter via the AuxRE. We show that interfering with *ARF5*/*MP*‐dependent auxin signaling attenuates miR390 expression in the transit‐amplifying compartment of the root meristem. Our results show that ARF5/MP regulates directly the expression of miR390 in the root meristem. We propose that ARF5, miR390, and the ta‐siRNAs‐regulated ARFs modulate the response of the transit‐amplifying region of the meristem to exogenous auxin.

## 1 INTRODUCTION

Growth of the *Arabidopsis thaliana* root is supported by its apical meristem. This root meristem consists of three main regions. The quiescent center (QC), formed by few cells that barely divide, is surrounded by stem cells which divide to form the different cell types that comprise the stereotypical Arabidopsis root. The proximal meristem, located shootward from the QC, is the region where stem cell progeny undergoes rapid, transit‐amplifying cell divisions that provide the necessary number of cells for organ growth (Scheres, 2007). Cells leaving the meristem undergo rapid cell elongation without division in elongation/differentiation zone (Heidstra & Sabatini, 2014). The size of the meristem is homeostatically regulated by matching the rates of cell production in the meristem and differentiation in the elongation/differentiation zone (Heidstra & Sabatini, 2014). This balance results from the interplay between auxin and cytokinin signaling (Dello Ioio et al., 2007, 2008). The transcriptional auxin signaling relies on Auxin/Indole‐3‐acetic acid (Aux/IAA) proteins that bind and inhibit auxin response factors (ARFs), DNA‐binding proteins in charge of regulating auxin‐dependent genes. ARFs bind DNA via an auxin response element (AuxRE) (Boer et al., 2014). In the presence of auxin, a complex is formed between Aux/IAA and TIR1/AFB triggering the poly‐ubiquitination and subsequent degradation of Aux/IAA, unlocking the ARFs (Paque & Weijers, 2016).

ARF5/MONOPTEROS (MP) plays an essential role in relaying the effects of auxin in multiple developmental contexts (Aida, Vernoux, Furutani, Traas, & Tasaka, 2002; Bhatia et al., 2016; Hardtke & Berleth, 1998; Przemeck, Mattsson, Hardtke, Sung, & Berleth, 1996; Smet et al., 2010). MP is essential for the formation of the embryo axis by specifying the root and vasculature. MP is expressed in the lower third of the early embryo and MP loss‐of‐function prevents the formation of the embryonic root (Weijers et al., 2006). During post‐embryonic development, 14 of the 23 ARFs present in Arabidopsis (Okushima et al., 2005) have been reported to be expressed in the primary root tip (Marin et al., 2010; Rademacher et al., 2011), including MP. Whereas full loss‐of‐function *mp* alleles leads to rootless embryos, in weak *mp* allele (*mp‐S319*), a root is formed but its growth is impaired (Cole et al., 2009). *ARF3* (Marin et al., 2010), *ARF2*,* ARF8*,* ARF10*,* ARF16, ARF17* (Rademacher et al., 2011) are expressed in the root meristem and their abundance controlled by endogenous small regulatory (s)RNAs (Kasschau et al., 2003; Poethig et al., 2006).

Micro (mi)RNA and trans‐acting small interfering RNAs (ta‐siRNAs) are endogenous small regulatory RNAs that regulate post‐transcriptionally the abundance of their target (Bologna & Voinnet, 2014) and control many processes in plants (Mallory & Vaucheret, 2006), in particular root development (Carlsbecker et al., 2010; Kasschau et al., 2003; Marin et al., 2010; Rodriguez et al., 2015; Stauffer & Maizel, 2014; Yoon et al., 2010, 2014; Yu, Niu, Ng, & Chua, 2015). Of special interest is the *TAS3* ta‐siRNA pathway, in which miR390 triggers the biogenesis of ta‐siRNAs by ARGONAUTE (AGO)7‐mediated cleavage of the non‐coding RNA *TAS3*. Subsequent recruitment of SUPPRESSOR OF GENE SILENCING 3 (SGS3) and RNA‐DEPENDENT RNA POLYMERASE 6 (RDR6) to the cleaved *TAS3*, results in the DICER‐LIKE 4 (DCL4)‐dependent production of 21‐nt secondary siRNAs called ta‐siRNAs targeting members of the *ARF* family (Bologna & Voinnet, 2014). The *TAS3* ta‐siRNA pathway is conserved across land plants and has been repeatedly employed to regulate ARFs abundance and confers sensitivity and robustness onto the auxin response (Plavskin et al., 2016). In Arabidopsis, ta‐siRNAs inhibit *ARF2*,* ARF3*, and *ARF4* and functions in the adaxial‐abaxial (top‐bottom) leaf polarity (Adenot et al., 2006; Fahlgren et al., 2006; Garcia, Collier, Byrne, & Martienssen, 2006; Hunter et al., 2006), heteroblasty (Hunter, Sun, & Poethig, 2003), biotic stress response (Cabrera et al., 2016), and lateral root outgrowth (Marin et al., 2010; Yoon et al., 2010). During lateral root formation, the *TAS3* pathway defines an autoregulatory network in which positive and negative feedback regulation of miR390 by ARF2, ARF3, and ARF4 ensures the proper expression of miR390 and maintains *ARFs* concentration in a range optimal for specifying the timing of lateral root growth (Marin et al., 2010; Yoon et al., 2010). The *TAS3* pathway appears thus to be integral to the control of auxin‐mediated lateral root growth. Interestingly, all components of the pathway are also expressed in the primary root meristem and miR390 has been shown to respond to auxin (Marin et al., 2010; Yoon et al., 2010). In addition, miR390 has been suggested to play a role in the auxin‐induced reduction of the meristem activity (Eliasson, Bertell, & Bolander, 1989; Mähönen et al., 2014; Thimann, 1937; Yoon et al., 2014). Yet, how auxin modulates the expression of miR390 is still unknown.

Here, we show that in the proximal meristem, cells of the transit‐amplifying compartment express miR390 and that auxin‐induced reduction of the proximal meristem impacts miR390 expression in the primary root. Ectopic expression of miR390 results in auxin hypersensitivity whereas loss‐of‐function results in hyposensitivity. We identify a short segment in the *MIR390a* promoter responsible for the expression of miR390 in the transit‐amplifying compartment. We show that ARF5/MP interacts directly with this segment via an AuxRE and controls miR390 expression in the proximal meristem. Our data show that miR390 plays a role in the control of the meristem size in response to exogenous auxin and that ARF5/MP is an essential regulator of its expression in the root meristem.

## 2 RESULTS

### 2.1 *MIR390a* is expressed in the root meristem where it modulates response to auxin

**Figure 1:**
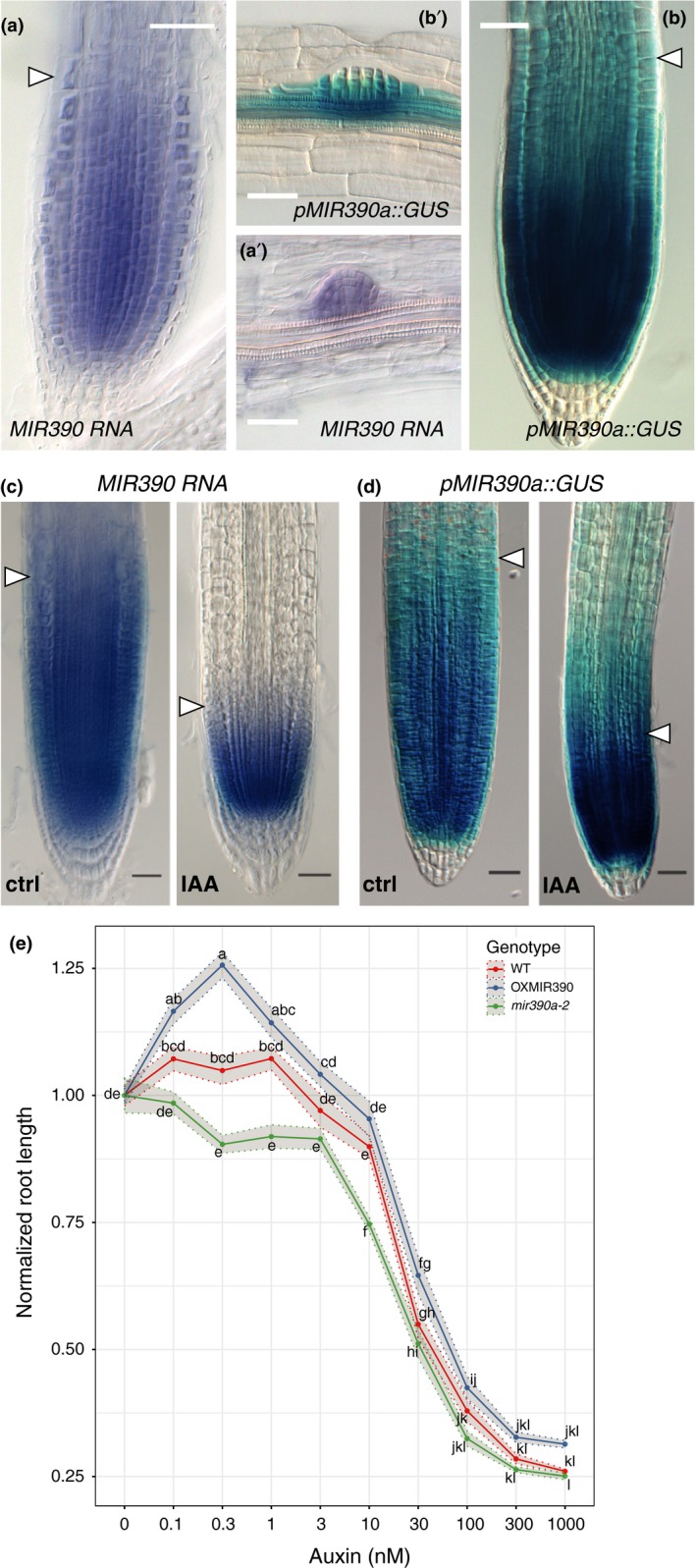
*MIR390a* is expressed in the root meristem and modulates response to auxin. (a, a’) Expression of miR390 detected by whole‐mount in situ hybridisation (WMISH) on wild type plants. miR390. (b, b’) expression pattern of the *pMIR390a::GUS* transcriptional reporter for *MIR390a*. miR390 and the *MIR390a* promoter are expressed in the primary root meristem (a, b) and in lateral root primordia (a’, b’). The arrowhead indicates the transition zone marking the end of the meristem. Images were taken at 5 days after germination (DAG) and scale bars are 25 μm. (c, d) Expression of miR390 by WMISH (c) and *pMIR390a::GUS* (d) in response to IAA treatment. Plants (5DAG) were treated by 1 μM IAA for 24 hr before fixation. Images were taken at 6DAG and scale bars are 25 μm. (e) Length of the primary root in response to IAA concentrations. Plants were grown for 3 days on control medium before transfer to medium containing the indicating amount of IAA for another 3 days. Root length after 6 days was normalised to its value in the absence of exogenous IAA. The lines represent the average root length of 20 plants and the shaded ribbons the standard error to the mean. Comparison between samples was performed using ANOVA and Tukey's HSD. Samples with identical letters do not significantly differ (α = 0.05)

A transcriptional reporter fusing 2.6 kb of sequence upstream of the *MIR390a* (At2g38325) stem loop to GUS (β‐glucuronidase) shows expression in the primary meristem and the lateral root primordia (Marin et al., 2010). The GUS pattern is identical to the one obtained by whole‐mount in situ hybridisation (WMISH) for miR390 (Figure 1a,b and Dastidar et al., 2016), in agreement with previous results showing that *MIR390a* is the only precursor expressed in the root (Dastidar et al., 2016; Marin et al., 2010). In the primary root meristem, we observe signal in the stem cell niche including the quiescent center, the vascular, epidermal, and cortex‐endodermal stem cells, but not in the columella stem cells. We also observe a graded expression in the transit‐amplifying compartment up to the transition zone, where the staining disappears. In the lateral root, the signal is marking the flanks of the primordium (Figure 1a’,b’ and Marin et al., 2010).

Upon treatment with 1 μM auxin (IAA) for 24 hr, the size of the meristem diminishes by reduction of the transit‐amplifying compartment (Figure 1c,d) with the concomitant reduction of the expression domain of miR390 (Figure 1c) and its promoter *pMIR390A* (Figure 1d). These data indicate that miR390 is expressed in the meristem, marking the transit‐amplifying compartment and that auxin and miR390 expression appear to be functionally connected.

The reduction of miR390 expression and accompanying upregulation of the ta‐siARF target ARF3 in the primary root meristem have been previously hypothesized to be involved in the inhibition of root growth induced by IAA (Yoon et al., 2014). To test whether miR390 is indeed involved in the regulation of the size of the transit‐amplifying compartment by auxin, we monitored the IAA inhibition of primary root growth in plants that either constitutively express miR390 (*p35S::MIR390A*, OXMIR390) or with reduced levels of miR390 (*mir390a‐2*) (Marin et al., 2010). Plants with lower levels of miR390 (*miR390a‐2*, Figure 1e) were more sensitive than wild type to low doses of IAA. Plants with mis‐expressed miR390 (OXMIR390, Figure 1e) had overall similar response to wild type, albeit with a mild trend toward increased primary root length in low (0.1–1 nM) IAA concentration. This result indicates that miR390 is required for the meristem response to IAA. Based on this result, we asked whether miR390 also controls primary root growth and meristem size in the absence of exogenous auxin. We did not observe any difference between *miR390a‐2* root length nor meristem size (Supporting Information Figure S1). Altogether, these results indicate that miR390 is expressed in the primary root meristem and is involved in the modulation of root growth in response to exogenous auxin.

### 2.2 A 36 bp‐long regulatory element is responsible for the expression of *MIR390a* in the root meristem

**Figure 2:**
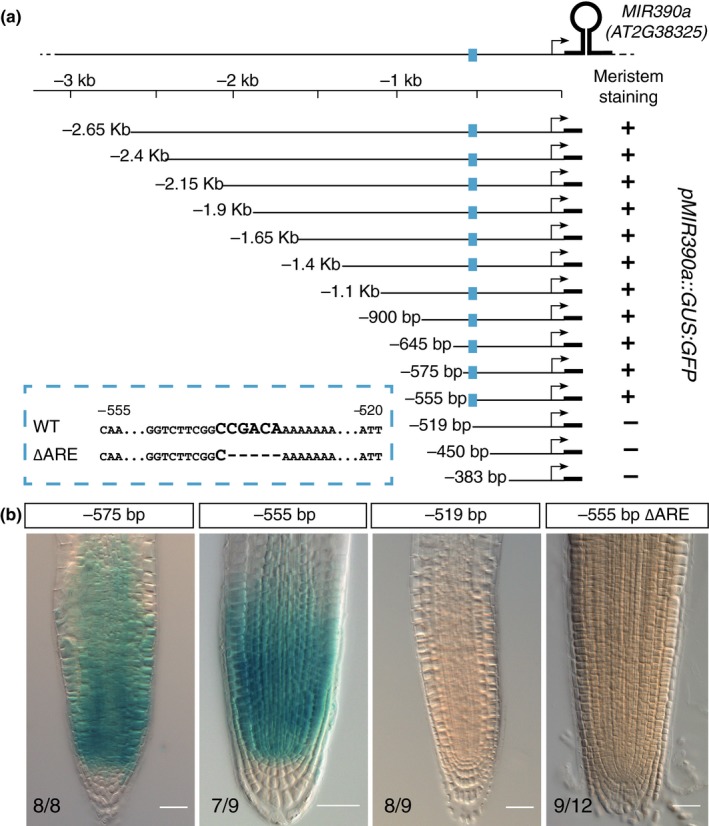
An auxin response element containing enhancer controls *MIR390a* expression in the root meristem. (a) Nested deletions in the region located upstream of the *MIR390a* stem loop region. The segment used to drive the expression of the GUS:GFP reporter is represented by a line, the *MIR390a* transcription start by an arrow and the stem loop by a thick line. The position of the primary root enhancer is indicated in blue and its sequence as well as the putative auxin response element (ARE) are shown in the dashed blue box. (b) Meristem expression of GUS reporter driven by the indicated segment of *MIR390a* promoter. The numbers indicate the proportion of independent primary transformants displaying the phenotype and scale bars are 25 μm

Given the role of miR390 in the meristem response to auxin, we sought to identify which region of the *MIR390a* promoter is responsible for the expression in the primary root meristem. For this, we generated nested 5′ deletions in the 2.6Kb fragment and fused these to a GUS reporter (Figure 2). For each construct, GUS staining was performed and the presence of signal in the meristem region was scored (*n* = 7–20 primary transformants). All reporters containing regulatory sequence up to position −555 bp from the transcription start site (+1) had the same expression pattern as the original 2.6 Kb long *pMIR390a::GUS* reporter (Figure 2a,b). On the contrary, reporters using 519 bp‐long or shorter fragments did not present any staining in the meristem (Figure 2a,b). This indicated that the 36 bp located between positions −555 and −519 is necessary for expression of the reporter in the primary root meristem. This sequence was dubbed the primary root regulatory element (PRE). Examination of the PRE revealed that it contains a single copy of a putative high affinity auxin response element (CCGACA, AuxRE) (Boer et al., 2014). To test the functional importance of this putative AuxRE to the activity of the PRE and to the expression of *MIR390a* in the root meristem, we deleted this motif in the context of the −555 bp reporter (−555∆ARE) and scored for the presence of GUS staining in the meristem of primary transformants. Whereas reporters using the control −555 reporter showed staining of the meristem (77%, *n* = 9), no expression was detected in the meristem of plants transformed with the −555∆ARE reporter (75%, *n* = 12). Taken together, these data indicate that expression of *MIR390a* in the primary root meristem is dependent on a single PRE and on the presence of a putative AuxRE therein.

### 2.3 A set of five ARFs interact with the PRE *ex planta*

**Figure 3:**
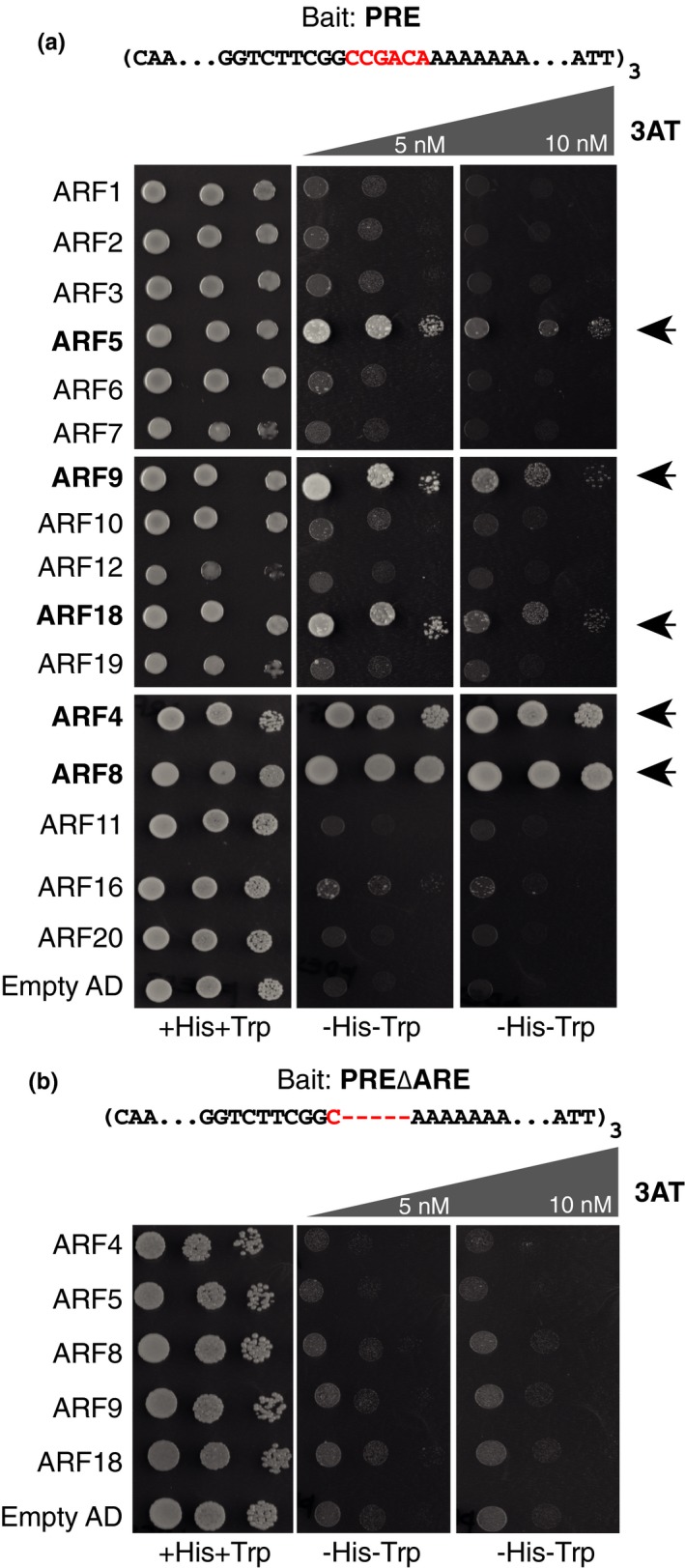
Five auxin response factors (ARFs) interact with *MIR390a* primary root enhancer (PRE) in yeast. (a) Yeast co‐expressing a trimerized version of the PRE as bait and a fusion between the indicated ARF and the Gal4 activation domain (AD) were grown on selective media in the presence or not of 3AT. ARF5, ARF9, ARF18, ARF4, or ARF8 interacted with PRE resulting in yeast growth on selective medium (arrowheads). (b) Yeast expressing as bait a trimerized version of the PRE lacking the auxin response element (ARE) did not grow on selective medium

The presence of a putative AuxRE in the PRE of *pMIR390a* leads us to seek which ARF could interact with this CCGACA motif. For this, we performed a targeted enhanced yeast one‐hybrid (Y1H) screening (Gaudinier et al., 2011) using a trimerized version of the PRE as a bait and a collection of 16 ARFs as preys. Yeasts expressing ARF4, 5, 8, 9, and 18 grew on selective medium lacking tryptophan and histidine in the presence of 10 mM 3AT, whereas growth was much reduced or undetectable in yeasts expressing any of the other ARFs or no ARF at all (Figure 3a). This result indicates that ARF4, 5, 8, 9, and 18 interact with the trimerized PRE. To test whether the interaction required the presence of the AuxRE motif, we repeated the yeast assay expressing this time as a bait a trimerized version of the PRE in which the putative AuxRE has been deleted (PRE∆ARE). In these conditions, yeast expressing ARF4, 5, 8, 9, and 18 were not able to grow (Figure 3b) indicating that interaction between these ARFs and the PRE requires the presence of the ARE.

### 2.4 ARF5/MP controls the expression of miR390 in the root meristem

**Figure 4:**
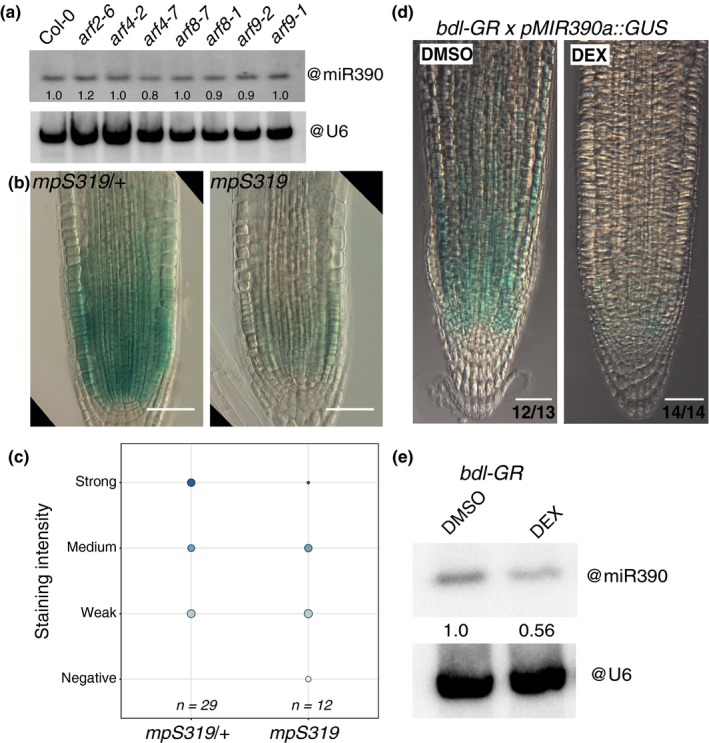
ARF5/MP controls the abundance of miR390 in the primary root meristem. (a) RNA gel blot analysis of 15 μg of root RNA from 7DAG wild type (Col) or the indicated *ARF* mutant plants hybridized with miR390. U6 snRNA served as a loading control, and numbers are the ratios of miR390 to U6 signal. (b) Expression of *pMIR390a::GUS* in heterozygous (*mpS319/+*) and homozygous (*mpS319*) *monopterous* mutants. Scale bars are 50 μm. (c) Distribution of the *pMIR390a::GUS* intensity in heterozygous (*mpS319/+*) and homozygous (*mpS319*) *monopterous* mutants. (d) Expression of *pMIR390a::GUS* in F1 of a cross between *pRBCS5A::bdl‐GR* and *pMIR390a::GUS* upon treatment with 10 μM dexamethasone (DEX) for 48 hr to induce the nuclear translocation of *bdl* and inhibition of MP activity or in control conditions (DMSO). The proportion of plants with the depicted signal is indicated. Scale bars are 50 μm. (e) RNA gel blot analysis of 15 μg of root RNA from 7‐day‐old *pRBCS5A::bdl‐GR* treated for 48 hr with 10 μM DEX or DMSO and hybridized with miR390. U6 snRNA served as a loading control, and numbers are the ratios of miR390 to U6 signal

To identify which of these five ARFs is a bona fide interactor of *pMIR390a* PRE *in planta*, we first examined which of them are expressed in the root meristem. For this, we used previously generated transcriptional reporters (for ARF4 and ARF8) and translational fusions (for ARF5, ARF9, and ARF18) (Marin et al., 2010; Rademacher et al., 2011). The expression pattern of the reporters showed that ARF4, ARF5, ARF8, ARF9, and ARF18 are expressed in the root meristem (Supporting Information Figure S2) in domains overlapping with the one of *MIR390a* and could regulate its expression. We then tested if the expression of miR390 is dependent on these ARFs. We quantified the abundance of miR390 by northern blot in plant mutants for the candidate interacting ARFs ARF4, ARF8, and ARF9 as well as ARF2 which did not show interaction with the PRE in yeast (Gutierrez et al., 2009; Hunter et al., 2006; Marin et al., 2010; Okushima et al., 2005; Tian et al., 2004). The levels of miR390 were unaltered between wild type and *arf2*,* arf4*,* arf8,* or *arf9* mutants (Figure 4a). Since strong alleles of *ARF5/MP* lead to defects in axis formation and patterning in embryos and rootless seedlings (Donner, Sherr, & Scarpella, 2009; Hardtke & Berleth, 1998; Okushima et al., 2005; Schlereth et al., 2010), we crossed the *pMIR390a::GUS* reporter line in the weak *arf5* allele *mpS319* (Cole et al., 2009) and performed GUS staining in the F2 progeny of the cross. Whereas GUS signal was observed at the root tip region of plants heterozygous for *mpS319*, no staining was detected in *mpS319* homozygous plants (Figure 4b,c). We also performed WMISH and RT‐qPCR for miR390 in heterozygous and homozygous *mpS319* seedlings. In both cases, levels of miR390 where strongly reduced in homozygous *mpS319* plants compared to heterozygous (Supporting Information Figure S3A,B). Together, these results suggest that ARF5/MP is required for miR390 expression in the root meristem.

To further test that ARF5/MP is necessary for miR390 expression, we monitored miR390 expression in the *pRBCS5A::bdl‐GR* background (Schlereth et al., 2010). In this line, a stabilised (and therefore dominant) version of the AUX/IAA BODENLOS/IAA12 which interacts with ARF5/MP is expressed as a GR fusion in the meristem. In the absence of dexamethasone (DEX), ARF5/MP activity is normal and consequently meristem organisation and root development are unaltered. Upon DEX treatment, the *bdl* version of IAA12 translocates to the nucleus and inhibits ARF5/MP‐mediated transcription. We first monitored the expression of the *pMIR390a::GUS* reporter in this inducible knockdown of ARF5/MP. Forty‐eight hours after DEX treatment, expression of the *pMIR390a::GUS* reporter was reduced in the root meristem without gross alteration of the organisation of the meristem supporting that ARF5/MP controls expression of the reporter (Figure 4d). We then asked whether the endogenous expression of miR390 was also altered when ARF5/MP is knocked down. We detected mature miR390 by in situ hybridisation (Supporting Information Figure S3C) and by northern blot (Figure 4e) in *pRBCS5A::bdl‐GR* plants treated or not treated by DEX for 48 hr. We observed a reduction of in situ signal at the root meristem (Figure 4e) and a 44% reduction of miR390 signal by northern blot upon DEX treatment (Figure 4f), confirmed by RT‐qPCR for miR390 (Supporting Information Figure S3D). Together, these data indicate that ARF5/MP is required for miR390 expression at the meristem.

### 2.5 ARF5/MP modulates transcription of *MIR390a* via the AuxRE in the PRE in plant cells

**Figure 5:**
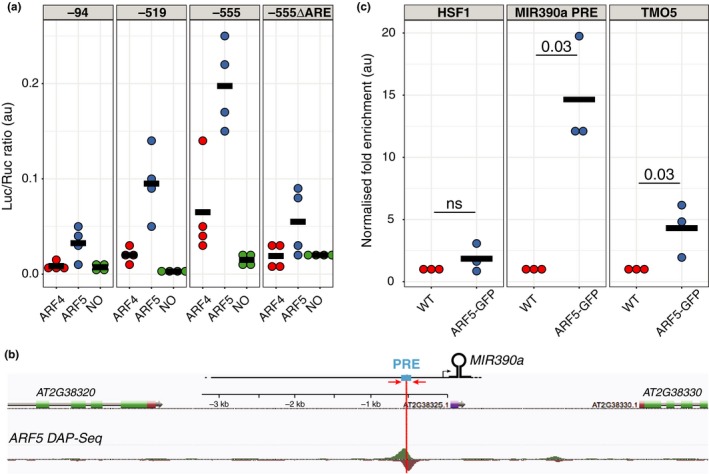
ARF5/MP activates the *MIR390a* promoter via the auxin response element located in the primary root enhancer. (a) Fusions between the VP16 domain and ARF4 (ARF4), ARF5 (ARF5) or empty plasmid (NO) were co‐expressed in tobacco leaves with different segment of the *MIR390a* promoter (−94 bp, −519 bp or −555 bp see Figure 2) driving the expression of the firefly luciferase (Luc). In the ‐555∆ARE promoter, a 5 bp deletion removes the ARE in the −555 bp segment (Figure 2). A 35S‐driven renilla luciferase (Ruc) is used to normalise activity (Luc/Ruc, expressed in arbitrary units). Each dot is a biological replicate and the horizontal bars the mean of the four replicates. (b) Annotated screen shot of genome browser at the *MIR390a* locus with a ARF5 DAP‐seq peak (O'Malley et al., 2016) located at the same position as the PRE (blue box). Position of the primers used for ChIP‐qPCR (C) is depicted by red arrows. (c) Enrichment of ARF5‐GFP at three loci was assessed by ChIP‐qPCR. Chromatin prepared from wild type (WT) or ARF5‐GFP plants was immunoprecipitated by an anti‐GFP antibody and enrichment of ARF5‐GFP in the region PRE of *MIR390a* (*MIR390a* PRE), in the second exon of TMO5 or upstream of the HSF1 loci was assessed by qPCR. Fold enrichment in the immunoprecipitated fraction over input fraction in ARF5‐GFP sample was normalised to the one in WT plants and expressed as arbitrary unit. Each dot is a biological replicate and the horizontal bars the mean of the three replicates. Statistical significance was evaluated by the Kruskal‐Wallis test and *p* values are indicated

We found that ARF5/MP interacts in yeast with *pMIR390a* via the AuxRE located in the regulatory element required for expression of miR390 in the root meristem and that, genetically, ARF5/MP is required for miR390 expression in this region. To test whether ARF5/MP is able to directly induce the *MIR390a* promoter in plant cells, we co‐expressed in tobacco leaves ARF5/MP with different fragments of the *MIR390a* promoter. To circumvent the need to trigger auxin‐mediated degradation of AUX/IAA to activate ARF5/MP, ARF5/MP was expressed as a fusion with the VP16 activation domain. Co‐expression of ARF5/MP‐VP16 with the 555 bp‐long fragment of *MIR390a* promoter fused to Luciferase (LUC) was sufficient for expression (Figure 5a). When ARF4‐VP16 or no ARF were co‐expressed, reduced (ARF4) or no significant expression of LUC could be detected (Figure 5a). Variants of the promoter in which the PRE is not present (519 bp and 94 bp‐long segments) or elimination of the AuxRE element in the 555 bp‐long one (−555∆ARE) reduced strongly the levels of LUC expression in the presence of ARF5/MP‐VP16 (Figure 5a). Together, these results indicate that ARF5/MP is able to stimulate transcription from the *MIR390a* as long as it contains the PRE and its AuxRE.

### 2.6 ARF5/MP interacts with the PRE of *MIR390a* in Arabidopsis

Mining of the Arabidopsis cistrome data (O'Malley et al., 2016) revealed the presence of a DAP‐seq peak for ARF5/MP ~550 bp upstream of *MIR390a* transcription start which corresponds exactly to the location of the PRE regulatory element we identified (Figures 5b and 2a). This further substantiates that ARF5/MP interaction with this regulatory element might be relevant. To validate that ARF5/MP interacts with *MIR390a* in Arabidopsis, we performed a chromatin immunoprecipitation assay (ChIP) using a functional ARF5:GFP fusion protein driven by the native ARF5 promoter (ARF5‐GFP) (Schlereth et al., 2010). Control immunoprecipitations were performed on Col‐0 (WT). Promoter fragments encompassing the PRE region were found to be ~15‐fold enriched in ARF5‐GFP over WT (Figure 5c, *p* = 0.03, Kruskal‐Wallis with *n* = 3 biological replicates). Under the same conditions, ARF5‐GFP was enriched ~5‐fold at the *TARGET OF MONOPTEROS5* (*TMO5*) locus (Schlereth et al., 2010), which served as a positive control. No enrichment was observed at the unrelated locus heat shock factor 1 (HSF1) (Figure 5c and Supporting Information Figure S3). This result indicates that ARF5/MP interacts with *MIR390a* promoter in the PRE region.

## 3 DISCUSSION

Here, we show miR390 is expressed in the transit‐amplifying compartment responsible for the radial patterning of the root and its growth. This expression is controlled by a single enhancer and the high affinity AuxRE it contains. We show that ARF5/MP interacts with this enhancer via the AuxRE in yeast and in plant cells and that in planta ARF5/MP binding is enriched in the region of *MIR390a* promoter containing the enhancer. By interfering with ARF5/MP‐dependent auxin signaling, we show that the expression of the miR390 is reduced, showing that ARF5/MP is necessary for miR390 expression in this compartment of the meristem.

Like their protein‐coding gene counterparts, transcriptional regulation of miRNA gene involves regulation by cis‐regulatory elements and trans regulators. Although hundreds of miRNA have been identified in plants, there is only a handful of studies linking expression of miRNA to specific transcription factors. An in silico approach has shown that cis‐regulatory elements corresponding to binding motifs for transcription factors such as LEAFY (LFY), ARFs, and AtMYC2 are overrepresented in the putative promoter region of MIRNA genes (Megraw & Hatzigeorgiou, 2010; Megraw et al., 2006) but the functional relevance of these enrichment has not been tested. Timing of phase transition and flowering relies on the sequential activation of the miR156/SPL and miR172/AP2 regulatory networks (Wang, Czech, & Weigel, 2009; Wu et al., 2009). The miR156‐regulated SPL9 and SPL10 transcription factors promote the expression of the miR156 in a feed‐forward loop as well as the sequential expression of miR172 to promote floral transition (Wang et al., 2009; Wu et al., 2009). Transcription of miR398 in Arabidopsis is induced in response to copper starvation and is involved in the degradation of mRNAs encoding copper/zinc superoxide dismutase (Sunkar, Kapoor, & Zhu, 2006). The transcription factor SPL7 is required for the expression of miR398 and other copper‐responsive miRNA (miR397, miR408, and miR857). SPL7 binds directly to GTAC motifs in the miR398 promoter in vitro, and these motifs were essential and sufficient for the response to copper deficiency in vivo (Yamasaki, Hayashi, Fukazawa, Kobayashi, & Shikanai, 2009). Our results indicate that miR390 is one of ARF5/MP target in the meristem. miR390 is the trigger of a regulatory network fine‐tuning the abundance of ARF2, ARF3, ARF4 and therefore ensuring sensitivity and robustness to auxin signaling in several developmental contexts (Adenot et al., 2006; Cabrera et al., 2016; Fahlgren et al., 2006; Garcia et al., 2006; Hunter et al., 2003, 2003; Marin et al., 2010, 2010; Yoon et al., 2010). This regulatory network is evolutionary conserved (Plavskin et al., 2016) and characterized by a retrocontrol of the ta‐siRNA‐controlled ARF on the expression of the miR390 (Marin et al., 2010; Plavskin et al., 2016). We identified ARF5/MP as an upstream regulator of miR390 on the root meristem. We do not exclude that additional ARFs may also regulate the expression of miR390. Whereas ARF5/MP is not a target of the miR390/TAS3 module, it is interesting that an ARF controls the expression of the miR390, suggesting that the miR390/TAS3/ARF regulatory network is an integral part of the regulatory networks mediating auxin response. It would be interesting to study whether in other developmental contexts where the miR390/TAS3/ARF regulatory network has been implicated such as lateral root and leaf patterning, similar network motif involving ARF5/MP, or other ARF have also been coopted to regulate miR390 expression. During lateral root formation, ARF3 and ARF4 are respectively repressor and inducer of miR390 (Marin et al., 2010). Whereas we identified ARF4 as interacting with the AuxRE present in the primary root enhancer in yeast, ARF3 was not. It would be interesting to map the cis‐regulatory motif(s) controlling the expression of miR390 in the lateral root to see whether an AuxRE is present and which ARF interacts with it.

Reduction of miR390 levels leads to hypersensitivity of the meristem to the inhibition of meristem growth induced by mild exogenous concentrations of auxin, whereas increased miR390 levels have opposite effects. miR390 therefore acts as a modulator of the effects of auxin on the size of the transit‐amplifying compartment. Previous work has hypothesized, based on the modulation of expression patterns, that the miR390‐*TAS3*‐*ARF3* module could control differentially the behavior of the meristem in response to exogenous auxin (Yoon et al., 2014). Here, we provide experimental evidences that levels of miR390 modulate the responsiveness of the meristem to exogenous auxin.

ARF2, ARF3 and ARF4, promote primary root growth (Marin et al., 2010) and are post‐transcriptionally regulated by miR390/TAS3. A mutation in miR390 does not dramatically alter neither the size nor the growth capacity of the meristem in the absence of exogenous auxin suggesting that the effects of the miR390/TAS3 module in the primary root may be buffered by additional control mechanisms. This is coherent with the absence of effect of gain and loss‐of‐function in *TAS3* on primary root length (Marin et al., 2010). At least one other miRNA‐controlled regulatory network has been involved in the control of root meristem size. miR396 is transcribed in the QC and columella where it represses a set of growth regulating factors (GRFs) which are transcription factors that promote cell division. miR396 ensures the exclusion of these GRFs from the stem cell niche and contributes to the transition between the stem cell niche and transit‐amplifying compartment of the root meristem (Bazin et al., 2013; Rodriguez et al., 2015). The miR396/GRF and miR390/TAS3/ARF3 regulatory networks both control leaf development (Adenot et al., 2006; Fahlgren et al., 2006; Garcia et al., 2006; Hunter et al., 2006; Rodriguez et al., 2010) and have been shown to genetically interact (Mecchia, Debernardi, Rodriguez, Schommer, & Palatnik, 2013). It would be interesting to investigate if both modules also interact in the root meristem.

## 4 MATERIALS & METHODS

### 4.1 Plant materials and growth conditions

*Arabidopsis thaliana* accession Col‐0 was used throughout this study. For transient assays, *Nicotiana benthamia*na was used. The *miR390a‐2* mutant (WiscDsLox440F06) was described in Marin et al. (2010). The *arf8‐1,arf8‐7,arf9‐1,arf9‐2, arf4‐7* mutants were described in (Gutierrez et al., 2009; Hunter et al., 2006; Marin et al., 2010; Okushima et al., 2005; Tian et al., 2004), *MP‐GFP (MP::MP‐GFP)* and *pRPS5A::bdl‐GR* in (Schlereth et al., 2010), *mpS319* in (Cole et al., 2009), the *pARF4::GFP, pARF8::GFP*,* ARF9:GFP*, and *ARF18:GFP* in (Marin et al., 2010; Rademacher et al., 2011). *Arabidopsis thaliana* was grown on soil in controlled plant rooms at 23°C under long day conditions (16 hr day length, LED illumination 150 mmol quanta m^−2^ s^−1^). *Nicotiana benthamiana* was grown on soil at 25°C with 16 hr day length (150 mmol quanta m^−2^ s^−1^). For in vitro growth on plates, seeds were surface sterilized with 70% EtOH+0.1% SDS for 10–15 mins followed by washing three times with 99% EtOH and sown out on 1/2 MS (Murashige‐Skoog) medium plates and 2.3 mM MES (pH 5.8) in 0.8% phytoagar. After stratifying the seeds in the dark (4°C) for 2–3 days, plates were placed in a vertical orientation inside growing chambers (23°C, 150 mmol quanta m^−2^ s^−1^). For chemical treatments, DEX (D4902‐1G; Sigma‐Aldrich) was stored as 30 mM stocks in DMSO and used at 10 μM for the indicated periods. For auxin sensitivity assays, plants were initially grown for 3 days before transferred onto fresh plates containing the indicated concentrations of IAA (I3750‐25G‐A; Sigma‐Aldrich).

### 4.2 Statistical analysis and plotting

All statistical analysis and plotting were performed with R (www.r-project.org) and plotting with the ggplot2 package (v3.0.0.900).

### 4.3 Analysis of root growth

For root elongation measurements, seedlings were grown vertically for 10 days. Starting from day 3 after germination until the end of the experiment at day 10, a dot was drawn at the position of the root tip. Finally, plates were scanned on a flatbed scanner, and the root length was measured over time with Fiji (Schindelin et al., 2012). Meristematic zone length was determined according to the file of cortex cells from confocal microscopy images after mPS‐PI treatment (Truernit et al., 2008). The meristematic zone was defined as the region of isodiametric cells from the QC up to the cell that was twice the length of the immediately preceding cell (Dello Ioio et al., 2008).

### 4.4 Cloning and generation of transgenic lines

The p35S::MIR390 construct was obtained by Gateway cloning by PCR amplification of the *MIR390b* stem loop (AT5G58465) cloning in pDONR201 and recombination in pAM506 (p35S::GateWay_RfA:term). Nested deletions in *pMIR390a* were obtained by Gateway cloning. Each fragment was amplified by PCR, cloned in pJLSmart, and recombined in pKGWFS7 (Karimi, Bleys, Vanderhaeghen, & Hilson, 2007). Deletion of the AuxRE in the −555 segment was obtained by gene synthesis and cloned as before. For the Y1H, the coding regions of ARF*4,8,11,16,17,20* were amplified from total seedling cDNA cloned in pDONR221 or pCR8 and Gateway cloned in AD‐2μ destination vector by a Gateway LR reaction (Gaudinier et al., 2011). Trimerised version of the 36 bp PRE bait (with and without ARE) was obtained by gene synthesis and cloned in the yeast 1 hybrid bait vectors, pMW#2 containing the *HIS3* reporter gene and integrated in the strain (YM4271) (Deplancke, 2004). VP16‐fusions for ARF4 and ARF5 were generated by GreenGate cloning (Lampropoulos et al., 2013) with the following modules pUB10 (A), VP16 (B), ARFs coding regions (C), HA‐tag (D), and UB10term (E) in pGGZ003. Fragments of *pMIR390a* were Gateway cloned upstream of LUC in pLUC_GW vector. All primers are listed in Supporting Information Table S1.

### 4.5 GUS (β‐glucuronidase) assay, in situ hybridization and microscopy

β‐glucuronidase activity was carried out at 37°C overnight (16 hr) using 2 mM ferri/ferrocyanide as described (Weigel & Glazebrook, 2002). After GUS staining, whole seedlings were cleared and mounted on 50% glycerol and detected by light microscopy using differential interference contrast (DIC) on a Zeiss Axio Imager M1 (Carl Zeiss, Göttingen, Germany) microscope using a Plan‐Apochromat 20X/1.4 NA objective. Whole‐mount in situ hybridizations for miR390 were performed exactly as described (Dastidar et al., 2016). Confocal laser scanning microscopy was performed throughout the study using a Plan‐Apochromat 20x, 0.8‐NA lens on a Leica SP8 or SPE microscopes. Roots were stained with 10 μg/ml propidium iodide for 2 min, rinsed, mounted in water, and visualized after excitation by an argon 488‐nm laser line. The fluorescence emission was collected from 590 to 700 nm (propidium iodide) and 496 to 542 nm (GFP).

### 4.6 RNA isolation and RNA blot

For RNA isolation, phenol‐chloroform extraction procedure was carried out as described(Marin et al., 2010). Small RNA northern blot analysis was performed as described (Marin et al., 2010). RNA gel blots were hybridized with the miR390 probe together with U6 probe as a loading control. Non‐saturated signals were quantified on a Fuji FLA 7000 scanner.

### 4.7 miRNA expression analysis via RT‐qPCR

Total RNA was isolated from root tissue as described above. Total RNA (2 μg) was treated with RNase‐free DNase I (Fermentas). First‐strand cDNA synthesis was performed using SuperScript II Reverse Transcriptase (Invitrogen). Five times diluted cDNA was used for amplification. A parallel reaction without reverse transcriptase enzyme was used as a control for genomic DNA contamination. Quantitative PCR was performed using a modified protocol to detect mature microRNA (Chen, 2005) with a BioRad thermocycler using SYBR Green I (Roche) to monitor double‐stranded DNA synthesis. The relative transcript level was determined for each sample and normalized (Livak & Schmittgen, 2001) using the PROTEIN PHOSPHATASE2A cDNA level (AT1G13320, athRef1; Czechowski, Stitt, Altmann, Udvardi, & Scheible, 2005). Melting curve analyses at the end of the process and “no template controls” were performed to ensure product‐specific amplification without primer‐dimer artefacts. Primer sequences are given in Supporting Information Table S1.

### 4.8 Dual‐luciferase reporter assay

Tobacco plants were infiltrated with the indicated combination of VP16‐fused ARF and *pMIR390a::LUC* constructs along a *p35S::renillaLuciferase*. Forty‐eight hours post infiltration detection of luciferases was performed with the dual‐luciferase reporter assay system (Promega) and a TECAN Infinite M1000 plate reader.

### 4.9 Yeast one‐hybrid assay

The eY1H protocol previously described (Gaudinier et al., 2011) was followed. Coding regions of *ARF 4, 8, 11, 16, 17, 20* were cloned in the laboratory whereas vector containing *ARF1, 2, 3, 5, 6, 7, 9, 10, 12, 18, 19* was obtained from S. Brady (UC, Davis, USA).

### 4.10 ChIP

ChIP experiments were performed as described in (Gendrel, Lippman, Martienssen, & Colot, 2005) with a few modifications. One to two grams of *A. thaliana* root tissue from 7‐day‐old wild type and MP‐GFP plants were used. Fixation and cross‐linking by 1% formaldehyde were performed twice for 10 mins under vacuum. Tissue was rinsed thoroughly in water, dried, frozen in liquid nitrogen, and ground. The resulting powder was resuspended in 30 ml of extraction buffer 1 for 10 min at 4°C before being filtered twice on 90 and 50 μm meshes then centrifuged at 3,000 g for 20 mins at 4°C. The pellet was resuspended in 300 μl extraction buffer 2 and centrifuged at 12,000 *g* for 10 mins at 4°C. The pellet was resuspended in 300 μl extraction buffer 3 and centrifuged at 16,000 *g* for 1 hr at 4°C. The resulting pellet was then lysed with 300 μl of nuclei lysis buffer and then sonicated for 10 mins. The debris was pelleted by centrifugation at 12,000 *g* for 5 mins at 4°C. At this point, 10 μl of the sample was set aside as “Input control” and the rest was used for immunoprecipitation after diluting 10 times with ChIP dilution buffer. The samples were divided in different tubes and incubated for 1 hr with gentle agitation with 40 μl of Protein A agarose beads (Invitrogen). After spinning down the beads, the pre‐cleared supernatant was incubated overnight with 0.5 μl anti‐GFP antibody (ChIP Grade; Abcam ab290) and Protein A agarose beads at 4°C with rotation. Beads were centrifuged at 3,800 *g* at 4°C for 30 s and successively washed in low salt wash buffer, high salt wash buffer, LiCl buffer, and TE buffer. Immune complexes were eluted from the beads by incubation in 250 μl elution buffer at 65°C for 15 mins with intermittent shaking. After beads were pelleted, proteins were reverse cross‐linked by adding 20 μl of 5M NaCl at 65°C overnight. All samples were treated with 2 μl of Proteinase K (10 mg/ml) and DNA extracted using Qiagen mini elution kit and dissolved in 40 μl of water. Fold enrichment at specific loci was quantified by qPCR on 1 μl of each sample with the respective primers, as ratio of the immunoprecipitated fraction over input in MP‐GFP normalised to the same ratio in WT. The primers used in the ChIP experiments are listed in Supporting Information Table S1.

## AUTHORS’ CONTRIBUTIONS

MGD, AS, IM, PRD, PVB, LB, and VJ performed the experiments and analyzed the results with AM. AM wrote the paper with contributions from MGD, AS, PVB, LB.

## CONFLICT OF INTEREST

The authors declare no conflict of interest.
