# Supplementary material for: ARF5/MONOPTEROS directly regulates miR390 expression in the Arabidopsis thaliana primary root meristem
Source: Plant Direct. 2019 Feb 5;3(2):e00116. doi: 10.1002/pld3.116 (PMC6508847; doi:10.1002/pld3.116)
Supplement: Supplementary file 1 [file PLD3-3-e00116-s001.pdf]

Supplemental Material

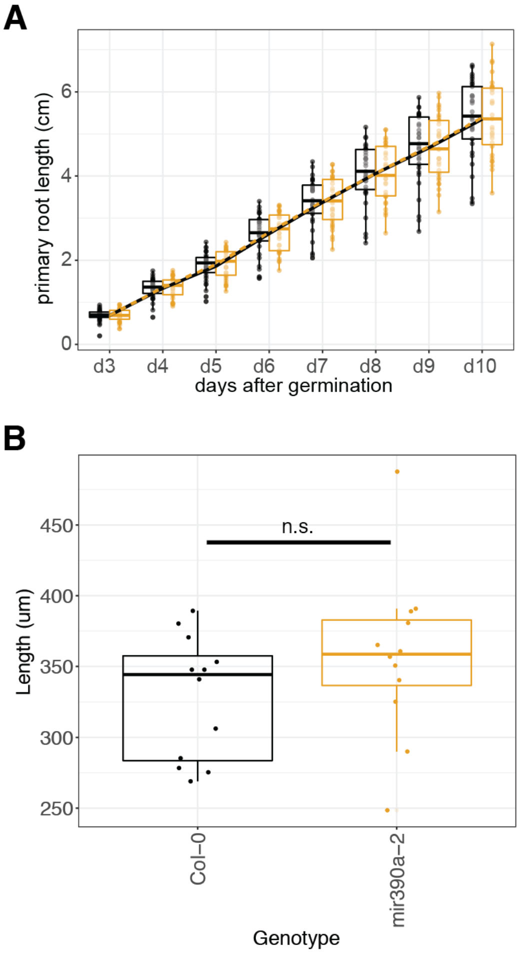

Supplemental Figure 1 (related to Figure 1) Root growth and meristem of *mir390a-2*

(A) Root elongation (cm) in wild type and *mir390a-2*. Box plots represent the distribution of 32 plants.

(B) Meristematic zone length (µm) in wild type and *mir390a-2*. Box plots represent the distribution of 12 plants.

In the box plots, the thick line represent the mean of the distribution, the box the 25th-75th interquartile range, the lower whisker the 5th quartile and the upper one the 95th quartile.

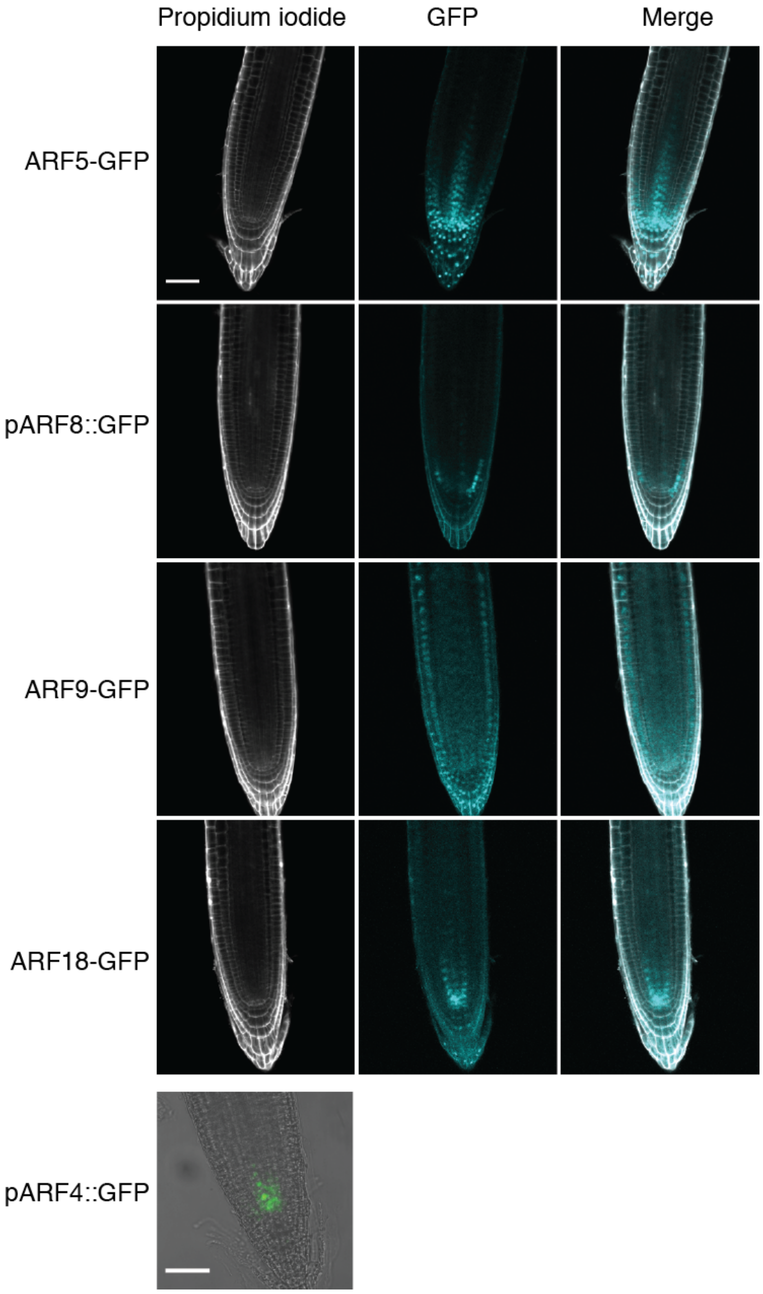

Supplemental Figure 2 (related to Figure 3) Expression of the PRE-interacting candidates ARFs in the root meristem.
Confocal sections of the indicated translational (ARF5-GFP, ARF9-GFP, ARF18-GFP) or transcriptional (pARF8::GFP, pARF4::GFP) fusions.

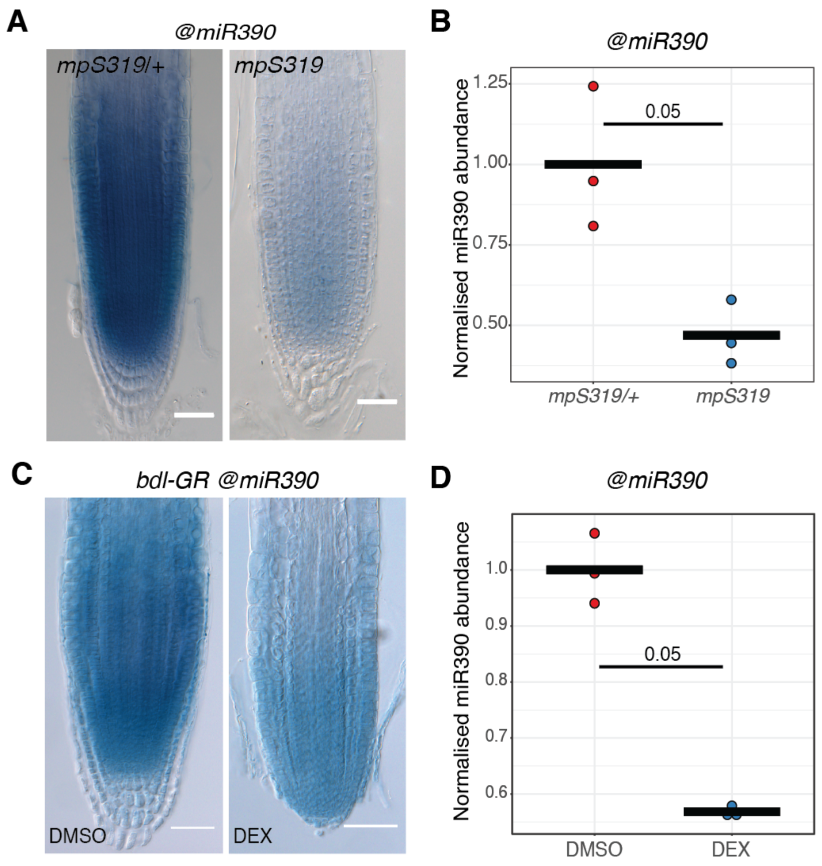

Supplemental Figure 3 (related to Figure 4). Reduction of miR390 levels upon inhibition of ARF5/MP.

(A) Expression of miR390 by WMISH in *mpS319* homozygous and heterozygous (*mpS319*/+). Images were taken at 6DAG and scale bars are 50µm.

(B) RT-qPCR analysis of miR390 levels in *mpS319* homozygous and heterozygous (*mpS319*/+) plants. Each dot represent the abundance of miR390 normalised to athRef1 (AT1G13320) in a biological replicate and the horizontal bars the mean of the three replicates. Statistical significance was evaluated by the Kruskal-Wallis test and p values indicated.

(C) Expression of miR390 by WMISH in *bdl-GR* plants treated with 10µM DEX or DMSO as control for 3 days. Images were taken at 6DAG and scale bars are 50µm.

(D) RT-qPCR analysis of miR390 levels in *bdl-GR* plants treated with 10µM DEX or DMSO as control for 24h. Each dot represent the abundance of miR390 normalised to athRef1 (AT1G13320) in a biological replicate and the horizontal bars the mean of the three replicates. Statistical significance was evaluated by the Kruskal-Wallis test and p values indicated.

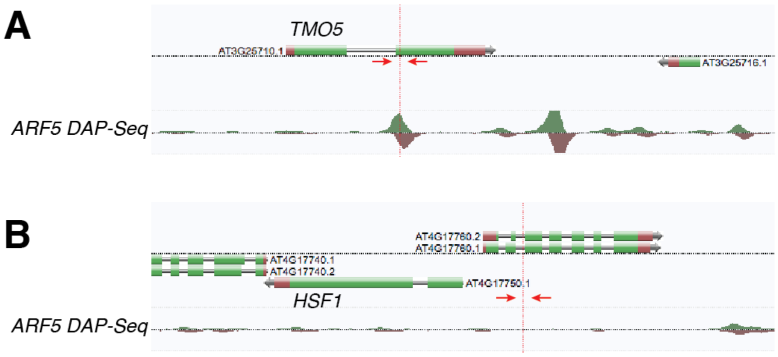

Supplemental Figure 4 (related to Figure 5). ARF5 DAP-Seq at TMO5 and HFS1 loci. Annotated screen shot of genome browser at the TMO5 (A, AT3G25710) and the HSF1 (B, AT4G17750) locis with a ARF5 DAP-seq peaks (O'Malley et al. 2016). Position of the primers used for ChIP-qPCR (Figure 5C) at each locus is depicted by red arrows.
