# Supplementary material for: ARF5/MONOPTEROS directly regulates miR390 expression in the Arabidopsis thaliana primary root meristem
Source: Plant Direct. 2019 Feb 5;3(2):e00116. doi: 10.1002/pld3.116 (PMC6508847; doi:10.1002/pld3.116)
Supplement: Supplementary file 3 [file PLD3-3-e00116-s003.pdf]

Response to the reviewers comments

Reviewer #1

Dastidar et al., demonstrate that ARF5 directly binds to a specific motif in the promoter of miR390, and upregulates miR390 expression in an auxin-dependent manner. This fits into what is already known, namely that auxin represses root elongation, miR390 targets TAS3 transcripts, and the resulting TASiARFs post-transcriptionally repress the ARF2, 3 and 4, transcription factors that are implicated in root elongation.

While the experiments are generally well executed, the manuscript does not provide much insight into root development nor the miR390/TAS3/ARF regulatory module in broader developmental contexts. The manuscript fails to put the arf5 phenotype into the context of the miR390/TAS3/ARF regulatory network. Neither the miR390 knock-down nor the overexpression line effected root elongation in the absence of auxin, which suggests that unlike ARF5, miR390 may not be that important for root development. What aspect of the arf5 developmental phenotype is due to lack of miR390 induction? This should be addressed. Where they do see an effect is upon auxin treatment (Fig 1E).

R/ We agree. As a mutation in miR390 does not dramatically alter neither the size nor the growth capacity of the meristem in absence of exogenous auxin, it suggests that the effects of the miR390/TAS3 module in the primary root may be buffered by additional control mechanisms. We have spent a long time looking for conditions (temperatures, nutrients limitation) that would unveil a statistically robust difference in root growth between WT and mir390a mutant, unfortunately without success.

The authors' conclusion (Line 144) that miR390 expression in the primary root meristem is modulated by exogenous auxin is not supported by the data. In fact they showed that expression persists in the root meristem as in control (Fig 1C). However, the size of the meristem is reduced with auxin application. The data more likely reflects the effects of ARF2,3,4 on the auxin response.

R/ We agree with the reviewer. Yet, our data show that miR390 marks the transient amplifying compartment and that auxin and miR390 expression are functionally connected. We have changed the sentence to “Altogether, these results indicate that miR390 is expressed in the primary root meristem and is involved in the modulation of root growth in response to exogenous auxin.”.

Furthermore, ARF4 is known to be upregulated in response to auxin, but there is no discussion of how this might affect the phenotypic readout. Therefore, additional experiments are required to elucidate the phenotypic contribution of miR390 regulation by ARF5, or potentially other ARFs.

R/ While we agree with the reviewer suggestion, such analysis falls out of the scope of the current manuscript that support that miR390 is a target of at least ARF5/MP. We have added a sentence in the discussion to make this point clear.

Y1H is known to produce false negatives, especially for transcription factors that have strong repression domains that can override the GAL4 activation domain. Therefore, this data alone cannot rule out the effects of other root expressed ARFs, such ARF2, 3 ARF6, 7, 19. Nulls of these alleles should be included in the analyses for miR390 expression in Fig 4A. The role of other ARFs should not be ruled out, especially as the expression pattern of ARF5 (Supp. Fig.2) does not mirror that of pmiR390:GUS (Fig1; Fig 4B). Furthermore, ARF18 was shown to bind the PRE in yeast, and to be expressed in the root, but was not examined further.

R/ We agree with the reviewer’s comments about the possibility that the Y1H may have overlooked other repressor ARF. We certainly do not exclude that other ARFs might contribute; yet, our results do support that miR390 is a target of at least ARF5/MP.

What does the DAP-seq data of the miR390 promoter (Fig 5B) look like for other ARFs in the PRE region - are they absent? Certainly miR390 levels in the mpS319 background as a positive control in Fig 4A to verify miRNA is being produced to lower levels.

R/ A focused peak DAP-Seq is only observed for ARF5/MP. The image below show that no defined peaks are present near the PRE for ARF2 and ARF16.

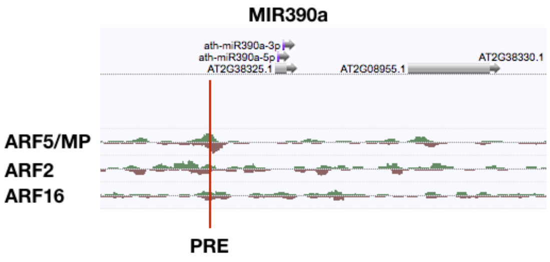

In the discussion the authors write 'It would be interesting to study whether in other developmental contexts where the miR390/TAS3/ARF regulatory network has been implicated such as lateral root and leaf patterning, similar network motif involving ARF5/MP or other ARF have also been co-opted to regulate miR390 expression' (Line 297). Given the genetic resources at hand these questions could be quite easily addressed.

R/ Although we agree with the reviewer suggestion, such analysis falls out of the scope of the current manuscript.

Minor:

In the discussion (line 320) it is also stated that miR396 non-cell autonomously represses GFFs in the root, but the referenced paper does not demonstrate nor imply non-cell autonomous behaviour of this miRNA.

R/ The sentence has been changed to “miR396 is transcribed in the QC and columella where it represses a set of GROWTH REGULATING FACTORs (GRFs) which are transcription factors that promote cell division. miR396 ensures the exclusion of these GRFs from the stem cell niche and contribute to the transition between the stem cell niche and transit amplifying compartment of the root meristem”

Fig 4. B, C legends says 'homozygous (mpS319) and heterozygous (mpS319/+) monopterous mutants', but the figures show the het first.
R/ The figure legend has been updated

Depending on where the 35S promoter is active, the MIR390 OE line may miss-express rather than over express.
R/ We have changed the wording on l136 to “Plants with mis-expressed miR390 (OXMIR390, Figure 1E) had overall similar response to wild type (…)”

Reviewer #2

This manuscript presents experiments showing that MP/ARF5 regulates the MIR390a promoter in the root meristem. While this seems correct and the work is well done, the overall interest of the work seems low because the mir390a mutant only has a mild phenotype in the presence of exogenous auxin, and no phenotype under normal growth conditions.

Specific points:

It seems possible that other ARFs in addition to MP also regulate the promoter, as they have not tested some obvious candidates such as ARF7 and ARF19 (exclusion based on a negative Y1H result seems overly stringent). The argument that ectopic stabilized bdl affects expression does not mean that it acts only through MP/ARF5, as BDL should interact with multiple ARFs.

R/ We agree with the reviewer and do not exclude that other ARFs may regulate the expression of miR390. The data presented here support that at least ARF5 is contributing to miR390 expression in the meristem.

Does exogenous auxin treatment reduce the domain of MP expression?
R/ In response to auxin, MP expression pattern is not drastically altered.

Are there growth conditions under which the mir390a mutant root meristem does have a phenotype, for example after wounding or stress?

R/ We have tested several growth conditions (temperatures, nutrients limitation) to uncover a statistically robust difference in root growth between WT and mir390a mutant, unfortunately without success .

In Figure 1E they present root length data relative to length in the absence of exogenous auxin. Perhaps meristem length would be more relevant. Also, are the baseline root lengths without auxin the same?

R/ As the root lengths of the gain and loss of function miR390 mutants are slightly, albeit statistically different from wild type, normalization of root length was necessary. The size of the meristem were not recorded in these experiments.

Do ARF2, ARF3, and ARF4 have roles in root meristem function? Such evidence might indicate how relevant miR390 regulation might be.
R/ *arf2*, *arf3* and *arf4* mutants have longer primary root than wild type (Marin et al. Plant Cell 10.1105/tpc.109.072553, supplemental material)

Reviewer #3

However, the primary and quite significant weakness of this paper is that the authors have no evidence that miR390 actually plays a role in root development. They show that miR390 affects the response of the root to exogenous auxin, however they were unable to find a root phenotype for a loss-of-function mutation in miR390. Consequently, the conclusion that "miR390...is necessary to maintain the size of the transit-amplifying region of the meristem" is not supported by the results presented in this paper.

R/ We have modified the abstract and the last paragraph of the introduction explicitly mentioning that miR390 affects the response of the root to exogenous auxin.
